# INDY—From Flies to Worms, Mice, Rats, Non-Human Primates, and Humans

**DOI:** 10.3389/fragi.2021.782162

**Published:** 2021-12-23

**Authors:** Dushyant Mishra, Kavitha Kannan, Kali Meadows, Jacob Macro, Michael Li, Stewart Frankel, Blanka Rogina

**Affiliations:** ^1^ Department of Genetics and Genome Sciences, School of Medicine, University of Connecticut Health Center, Farmington, CT, United States; ^2^ Department of Biology, University of Hartford, West Hartford, CT, United States; ^3^ Institute for Systems Genomics, School of Medicine, University of Connecticut Health Center, Farmington, CT, United States

**Keywords:** *Indy*, SLC13A5, citrate transporter, aging, longevity gene, calorie restriction

## Abstract

*I’m Not Dead Yet (Indy)* is a fly homologue of the mammalian SLC13A5 (mSLC13A5) plasma membrane citrate transporter, a key metabolic regulator and energy sensor involved in health, longevity, and disease. Reduction of *Indy* gene activity in flies, and its homologs in worms, modulates metabolism and extends longevity. The metabolic changes are similar to what is obtained with caloric restriction (dietary restriction). Similar effects on metabolism have been observed in mice and rats. As a citrate transporter, INDY regulates cytoplasmic citrate levels. *Indy* flies heterozygous for a P-element insertion have increased spontaneous physical activity, increased fecundity, reduced insulin signaling, increased mitochondrial biogenesis, preserved intestinal stem cell homeostasis, lower lipid levels, and increased stress resistance. Mammalian *Indy* knockout (*mIndy*-KO) mice have higher sensitivity to insulin signaling, lower blood pressure and heart rate, preserved memory and are protected from the negative effects of a high-fat diet and some of the negative effects of aging. Reducing *mIndy* expression in human hepatocarcinoma cells has recently been shown to inhibit cell proliferation. Reduced *Indy* expression in the fly intestine affects intestinal stem cell proliferation, and has recently been shown to also inhibit germ cell proliferation in males with delayed sperm maturation and decreased spermatocyte numbers. These results highlight a new connection between energy metabolism and cell proliferation. The overrall picture in a variety of species points to a conserved role of INDY for metabolism and health. This is illustrated by an association of high *mIndy* gene expression with non-alcoholic fatty liver disease in obese humans. *mIndy* (*mSLC13A5*) coding region mutations (e.g., loss-of-function) are also associated with adverse effects in humans, such as autosomal recessive early infantile epileptic encephalopathy and Kohlschütter−Tönz syndrome. The recent findings illustrate the importance of *mIndy* gene for human health and disease. Furthermore, recent work on small-molecule regulators of INDY highlights the promise of INDY-based treatments for ameliorating disease and promoting healthy aging.

## Introduction

The *I’m Not Dead Yet (Indy)* gene encodes a plasma membrane citrate transporter whose physiological role is highly conserved across species (Rogina et al., 2000; Knauf et al., 2002; Knauf et al., 2006; Rogina 2017). Reduced *Indy* gene activity in flies and reduced expression of *Indy* homologues in worms extends longevity by affecting metabolism in a manner similar to calorie restriction (CR) (Fei et al., 2004; Neretti et al., 2009; Wang et al., 2009; Rogers and Rogina 2014; Schwarz et al., 2015). The role of *Indy* in metabolism and longevity was first described in flies (Rogina et al., 2000). INDY was subsequently shown to be a citrate transporter in flies, worms, and mammals (Inoue et al., 2002a; Knauf et al., 2002; Inoue et al., 2004; Knauf et al., 2006). INDY has high sequence similarity to the mammalian transporter mINDY, also referred as mSLC13A5. However, the two transporters differ in their substrate affinity and transporting mechanism, which is discussed in detail in the next section (Inoue et al., 2002b; Knauf et al., 2002; Inoue et al., 2004; Knauf et al., 2006; Kopel et al., 2021). Intracellular citrate plays a key role in the energy homeostasis of a cell by affecting glycolysis, the TCA cycle, fatty acid synthesis and gluconeogenesis. Intracellular citrate levels are regulated by INDY and the mitochondrial citrate transporter *SLC25A1*, also called the citrate/isocitrate carrier (CIC) (Ferramosca and Zara, 2014). INDY transports extracellular citrate into cells and CIC exports citrate from mitochondria. INDY and mINDY are most highly expressed in tissues regulating systemic metabolism. In flies, INDY is predominantly expressed on the plasma membrane of midgut cells, fat body cells, and oenocytes (Rogina et al., 2000; Knauf et al., 2002). The fat body and oenocytes are analogous to human adipose tissue and liver, respectively (Ugur et al., 2016). In mammals, mINDY is primarily expressed in liver, testis and brain, (Inoue, et al., 2002a; von Loeffelholz et al., 2017), consistent with its roles in metabolic regulation and cell proliferation.

Here we review studies in mice, rats, tissue culture, non-human primates, and humans that confirm a highly conserved role of *Indy* in metabolism and cell proliferation (Rogina 2017). Genetic or pharmacological reduction of *Indy* activity confers benefits and prevents the negative effects of a high-fat diet or age (von Loeffelholz et al., 2017). Strikingly similar effects of *Indy* reduction on cellular metabolism and proliferation were observed in flies and hepatocarcinoma cells. In flies, *Indy* reduction in the midgut modifies metabolism, decreases ROS production, and reduces energy available for intestinal stem cell proliferation, thereby preventing an age-associated increase in intestinal stem cell proliferation (Rogers and Rogina 2014). Similarly, reduction of *mIndy* in human hepatocarcinoma cells inhibits cell proliferation by affecting metabolism and reducing energy available for cell division (Li et al., 2017). Recent studies describe the novel role of INDY in sperm maturation and food uptake, which is mediated by the close proximity of the fly testes and a specific region of the fly midgut (Hudry et al., 2019). Fly testes release the cytokine Upd1, which activates JAK/STAT signaling in enterocytes and regulates sex-specific differences in sugar-related gene expression. Citrate is released by INDY from enterocytes and is taken up by testes cells, where it mediates spermatocyte maturation. Citrate also mediates inter-organ signaling leading to increased food uptake (Hudry et al., 2019). Increased *mIndy* levels are associated with obesity and insulin insensitivity in patients with non-alcoholic fatty liver disease (NAFLD) (von Loeffelholz et al., 2017). Moreover, mutations in the *mIndy* coding region and loss of *mINDY* function in humans are linked to early infantile epileptic encephalopathy and Kohlschütter−Tönz syndrome, which are autosomal recessive diseases characterized by epilepsy, psychomotor delay, intellectual disability, and moderate gastrointestinal and pulmonary defects (Thevenon et al., 2014; Hardies et al., 2015; Klotz et al., 2016; Brown et al., 2021). Differences between beneficial and mild phenotypes associated with deletion of *mIndy* in mice and deleterious effects associated with the presence of two copies of mutations in the coding region of human mINDY, could be explained by species-specific differences in transporting characteristics, tissue-specific mINDY abundance and the cell-specific role of citrate in metabolism (Kopel et al., 2021). *Indy* research is a striking example of how studying flies can reveal novel genes of vital importance to humans (Helfand and Rogina, 2003a; Helfand and Rogina, 2003b). In addition to identifying this gene, flies and other model systems have revealed a previously unknown mechanism for regulating metabolism, homeostasis, health and longevity involving cytoplasmic citrate levels.

### Biochemical and Biophysical Properties of INDY (SLC13A5) Transporter

The *Indy* gene is a tricarboxylic acid (TCA) transporter first described in *Drosophila melanogaster* (Rogina et al., 2000). Studies by Inoue et al. and Knauf et al. identified *Indy* as a Na^+^-independent transporter for several TCA cycle intermediates with the highest affinity for citrate (Knauf et al., 2002; Inoue et al., 2002b; Inoue et al., 2004; Knauf et al., 2006). Functional studies using INDY-expressing *Xenopus* oocytes showed that INDY mediates efflux of [^14^C]citrate from oocytes in exchange for succinate. Additional studies have shown exchange of citrate for citrate and citrate for oxaloacetate. Furthermore, efflux of [^14^C]succinate from INDY-expressing oocytes could be stimulated by addition of citrate, α-oxaloglutarate, and fumarate. These experiments identified INDY as an anion exchanger that mediates the exchange of citrate for dicarboxylate and a proton. This transport is electroneutral and pH-dependent (Knauf et al., 2006).

Mammalian INDY (mINDY) from mice, rats, and humans is a Na^+^-dependent transporter with high affinity for citrate, in contrast to the Na^+^-independent nature of fly INDY (Inoue, et al., 2002a; Inoue, et al., 2002b; Inoue et al., 2004), and is called NaCT (Na^+^-coupled citrate transporter). The affinities of fly INDY and rat mINDY for citrate are similar. mINDY is a member of the DASS (divalent anion symporter) transmembrane protein family. This family has five members: SLC13A2 (NaDC1), SLC13A5 (NaCT or mINDY), and SLC13A3 (NaDC3), which predominantly cotransport di- and tri-carboxylates, and SLC13A1 and SLC13A4, which transport sulphate. DASS proteins function with an “elevator mechanism” wherein the transport domain carrying the carboxylate ion moves between inward and outward states across the cell membrane while attached to a scaffold domain (Mulligan et al., 2016; Sauer et al., 2021). Human and rat mINDY proteins function similarly, and have amino acids sequences exhibiting 77% identity and 87% similarity. However, there are differences in the affinity of the two transporters to citrate. The rat/mouse transporter is a high-affinity and low-capacity type, while the human mINDY (mSLC13A5) transporter is a low-affinity and high-capacity type (Kopel et al., 2021). This difference in affinities can be attributed to the various structural differences that exist between the two proteins. The rat/mouse transporters are completely saturated under physiological conditions with plasma citrate concentrations of 150–200 μM and will not respond to any further increase in circulating citrate levels. In contrast, human transporters are not saturated and therefore can respond to increases in citrate levels (Inoue et al., 2002b; Inoue et al., 2004). These differences in transport kinetics could lead to different outcomes associated with lack of mINDY in *mIndy* knock-out (*mIndy-KO*) mice or mutations in the citrate transporter in humans.

The first INDY crystal structure was determined for *Vibrio cholerae* INDY (VcINDY), a related transporter in bacteria, which shares only 26–33% sequence identity with its mammalian counterpart (Mancusso et al., 2012). The Ganapathy group provided a new model of the INDY protein structure using the Robetta pipeline, which attempts to explain the cause of detrimental effects of mutations in *mIndy* (*mSLC13A5*) in neonatal patients (Sauer et al., 2021). This model prediction is based on modeling of known structures of VcINDY and YdaH transporters (a transporter from AbgT transporter family in *Alcanivorax borkumensis*) and therefore could be significantly different that the actual structure of human mINDY. Sauer et al. (2021) also recently described the structure of the human citrate transporter mINDY in complex with citrate using cryo-electron microscopy (cryo-EM). The authors showed that mINDY forms a homodimer and consists of scaffold and transport domains. The mINDY-citrate complex confirms the Na-dependent binding of mINDY to citrate, with a ratio of four Na^+^ ions to one citrate. The precise positions of two out of four Na binding sites were identified, and these positions coincided with previous findings that Na^+^ affinities were reduced when mutations were generated in the surrounding residues (Schlessinger et al., 2014). The locations of the remaining two Na binding sites (Na3 and Na4) were not apparent in the structure. They observed that citrate was bound in a basic pocket between the Na1 and Na2 binding sites. The two Ser-Asn-Thr (SNT) motifs and surrounding residues are responsible for binding citrate. Several studies have shown that mutations in these regions led to reduced transport activities (Klotz et al., 2016). Several missense mutations that affect residues near the citrate binding sites in mINDY (mSCL13A5) cause epilepsy suggesting that these mutations might be interfering with citrate binding activities (Hardies et al., 2015).

### *Indy,* Metabolism and Longevity

**FIGURE 1:**
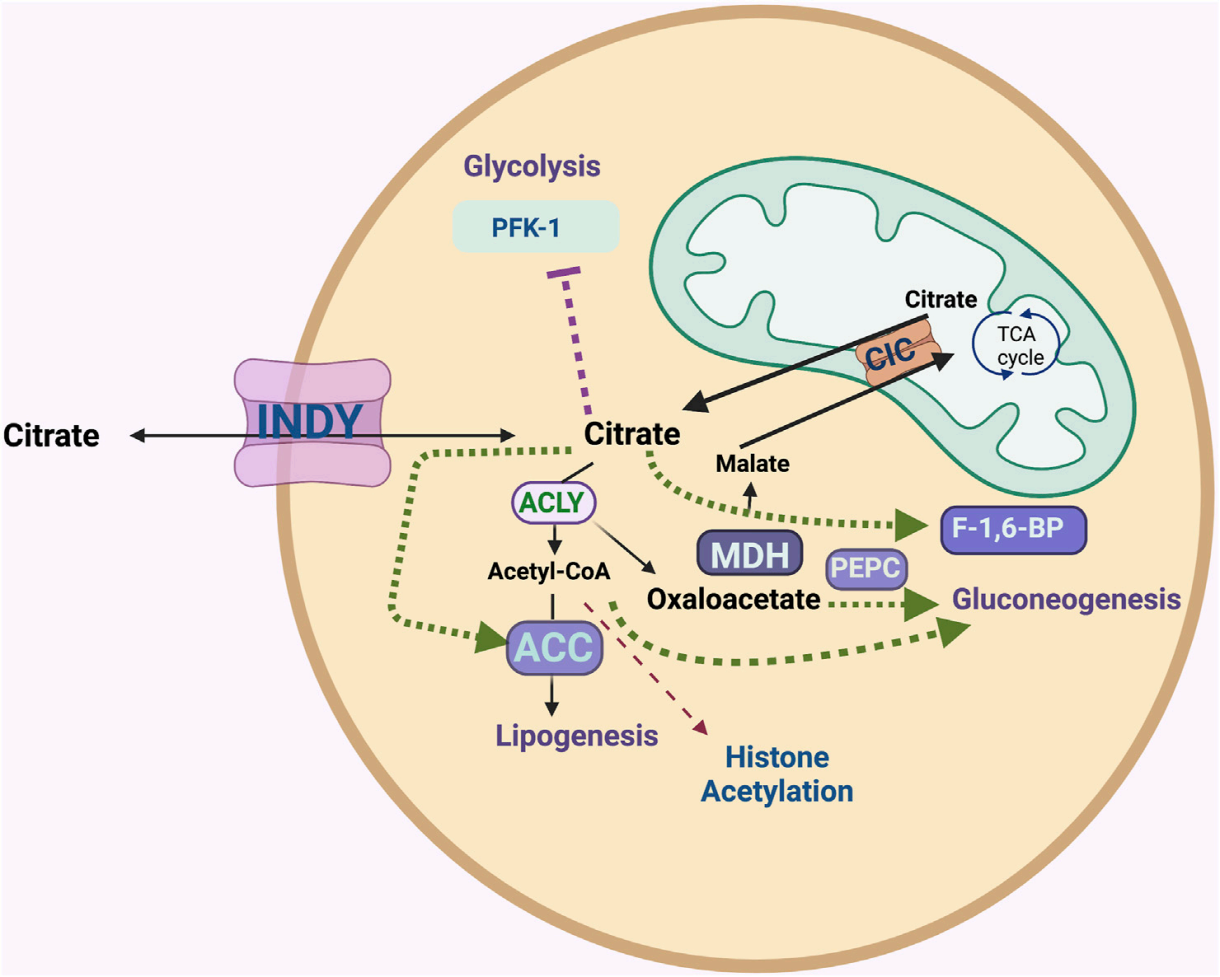
The INDY plasma membrane citrate transporter affects metabolism of several energy-related pathways. INDY as an exchanger of the TCA cycle intermediates between the cell and circulatory system affects cytoplasmic citrate levels. ATP-citrate lyase (ACLY) breaks down citrate to acetyl-CoA and oxaloacetate; the latter can be used in gluconeogenesis via phosphoenolpyruvate carboxylase (PEPC) or be converted to malate by malate-dehydrogenase (MDH). Acetyl-CoA carboxylase (ACC) catalyzes the first step of conversion of acetyl-CoA to fatty acids and cholesterol (lipogenesis). Acetyl-CoA plays a role in histone acetylation. Citrate inhibits glycolysis as an allosteric inhibitor of rate-limiting enzyme phosphofructokinase-1 (PFK-1). Citrate stimulates gluconeogenesis by allosterically activating fructose-1,6-bisphosphatase (F-1,6-BP). Excess citrate is transported from the mitochondria into the cytoplasm via the citrate/isocitrate carrier; CIC.

Citrate is a central metabolite in the TCA cycle. It is predominantly generated in the mitochondrial matrix from oxaloacetate and acetyl CoA. Its role in the TCA cycle is to produce NADH and FADH2, which ultimately produces ATP. Excess citrate is transported from the mitochondria into the cytoplasm by SLC25A1 (also named citrate/isocitrate carrier; CIC), which transports citrate out and brings malate into the mitochondria (Ferramosca and Zara, 2014). In the cytoplasm, citrate plays major roles in glycolysis, gluconeogenesis, and fatty acid and cholesterol biosynthesis, as well as acetylation of histones in the nucleus (Figure 1). INDY has different tissue expression patterns in different organisms, perhaps reflecting specialized needs for cytoplasmic citrate. For example, in neurons, citrate is involved in the production of several neurotransmitters. In addition to cellular citrate, high concentrations (150–200 μM) of citrate circulate in plasma. Circulating citrate is taken up by cells expressing INDY homologs.

In flies, reduction of *Indy* mRNA and INDY protein levels was originally studied using a P-element insertion in the first intron of the *Indy* gene, upstream of the putative transcriptional start site or the putative translational start site (Rogina et al., 2000; Knauf et al., 2002; Rogina and Helfand 2013; Rogers and Rogina 2014). While the presence of a P-element in the non-coding region affects transcription, leading to lower *Indy* mRNA and INDY protein levels, the protein remains wild type. Multiple independent *Indy* insertion alleles were subsequently identified, starting with the *Indy* ^*206*^, *Indy* ^*302*^ and *Indy* ^*159*^ alleles but expanding to include numerous additional P-element insertions; all of these alleles reduce *Indy* gene activity and confer longer lifespans. Exact excision of P-elements from several *Indy* alleles reverted longevity to control levels (Rogina et al., 2000; Rogina and Helfand, 2013; Rogers and Rogina, 2014). Some of these additional *Indy* alleles are intronic P-element insertions (Rogina and Helfand 2013; Rogers and Rogina, 2014). In both *Drosophila* and mice, lifespan is markedly influenced by genetic background, as would be expected for a complex phenotype affected by many pleiotropic genes and epistasis (Frankel and Rogina, 2021). *Indy* alleles produce longevity extension in several different genetic backgrounds, including the *Canton-S* wild type strain, the *Hyperkinetic*, *Shaker*, *drop-dead* strains, and the short- and long-lived lines created by Luckinbill (Luckinbill and Clare, 1985; Rogina et al., 1997; Rogina et al., 2000; Rogina and Helfand, 2013). Longevity was observed with the *Indy* ^*206*^/+ allele after 10 generations of backcrossing into the *yw* genetic background but not after backcrossing into the *w* ^*1118*^ background (Toivonen et al., 2007; Wang et al., 2009). The effect of CR upon lifespan, in both flies and mice, is also affected by genetic background (Frankel and Rogina, 2021). CR has a marginal effect upon lifespan in the *w* ^*1118*^ background. The lack of an effect of *Indy* ^*206*^/+ on lifespan in the *w* ^*1118*^ background is likely connected to the lack of a CR response in the same background (Toivonen et al., 2007; Wang et al., 2009). Longevity extension was also observed in natural populations of flies heterozygous for the *Hoppel* transposon insertion variant in the first intron of the *Indy* gene (Zhu et al., 2014). Flies heterozygous for the *Hoppel* element have lower *Indy* mRNA levels, higher fecundity and have preserved heterozygosity under laboratory conditions confirming fitness advantages. Physiological changes in *Indy* ^*206*^/+ flies are similar to those observed in calorie-restricted flies: increased mitochondrial biogenesis mediated by increased expression levels of the transcription factor *PGC1/Spargel*, reduced reactive oxygen species (ROS) production, reduced oxidative damage, increased resistance to oxidative stress, increased spontaneous physical activity, reduced insulin/insulin-like growth factor (IIS) signaling, metabolic changes similar to CR including lower lipid levels, and preserved intestinal stem cell homeostasis (Rogina et al., 2000; Bross, et al., 2005; Parashar and Rogina, 2009; Wang et al., 2009; Neretti et al., 2009; Frankel and Rogina 2012; Rogers and Rogina 2014). Reduction of INDY homolog expression in worms and mice also resembles the metabolic effects of CR. Worms in which RNAi-mediated silencing reduces *Indy* mRNA levels are smaller in size, have reduced lipid levels, and live longer (Fei et al., 2004; Schwarz et al., 2015). While the effect of mINDY reduction on the lifespan of mice has not been reported, *mINDY* ^*−/−*^ mice (also referred as *mIndy-KO*) have increased energy consumption, higher locomotor activity, decreased plasma glucose, and decreased insulin levels (Birkenfeld et al., 2011). On a high fat diet, there is reduced hepatic lipogenesis, increased mitochondrial biogenesis, enhanced hepatic fatty acid (FA) oxidation, protection from obesity, and preserved insulin sensitivity (Birkenfeld et al., 2011). Further support that *Indy* reduction mimics CR is provided by similarities in transcriptional changes found in the livers of *mINDY* ^*−/−*^ mice and CR mice (Birkenfeld et al., 2011). Similarly, adult rats kept on a high-fat diet have lower hepatic lipid levels and increased insulin sensitivity when INDY was reduced in the liver with antisense oligonucleotides (Pesta et al., 2015). Taken together, studies in flies, worms, and mice, show close parallels between *Indy* reduction and CR.

It is important to note that under normal laboratory conditions, longevity extension in *Indy* ^*206*^ */+* and *Indy* ^*302*^/+ flies is not associated with physiological tradeoffs: *Indy* ^*206*^ */+* and *Indy* ^*302*^/+ flies have the same metabolic rate, the same maximal flight velocity, the same food uptake and negative geotaxis, and even higher fecundity compared to controls (Marden et al., 2003; Wang et al., 2009). Aged on a high calorie diet, *Indy* ^*206*^ */+* flies don’t gain weight (Wang et al., 2009). Consistent with being in a state of CR, *Indy* ^*206*^ */+* and *Indy* ^*302*^/+ female flies aged on a low-calorie diet have lower fecundity compared to control flies on a CR diet (Marden et al., 2003). Longevity studies provide further support that *Indy* reduction creates CR: maximal effect on longevity was observed in *Indy* ^*206*^/+, *Indy* ^*302*^/+ and *Indy* ^*159*^/+ flies with mean lifespan extended between 80–100% and maximal life-span extended by about 50% (Rogina et al., 2000). Further *Indy* reduction, such as in *Indy* ^*206*^ */Indy* ^*206*^ homozygous flies, increases longevity by only 20% (Rogina et al., 2000; Rogina and Helfand, 2013). Longevity studies in flies raised on food with different calorie contents showed that *Indy* and CR’s effects on longevity interact. First, the levels of *Indy* mRNA are reduced in wild type flies on a CR diet (Wang et al., 2009). Longevity of *Indy* ^*206*^ */+* is greatly extended on a high calorie diet and standard diet, however, it is not further extended by CR. Longevity of *Indy* ^*206*^ */Indy* ^*206*^ homozygous flies is extended on a high calorie diet, to a lesser extent on a standard diet but it is reduced on a low-calorie diet, most likely due to starvation (Marden et al., 2003; Wang et al., 2009).

### *Indy* as a Metabolic Regulator: Effects of Age, Diet, Hormones, and Pharmacological Treatments on *Indy* mRNA Expression Levels

INDY plays a key role in metabolic regulation by affecting cytoplasmic citrate levels. This role has been underscored by studies showing that cell energy requirements affect *Indy* expression levels. For instance, *mIndy* levels in primary rat hepatocytes are up-regulated by glucagon, which is released during early starvation. Glucagon binds to the cAMP-dependent and cAMP-responsive element binding protein (CREB)-dependent binding site in the promoter region of *mIndy*, thereby increasing *mIndy* expression (Neuschäfer-Rube et al., 2014). This leads to increased citrate uptake and fatty acid synthesis in primary rat hepatocytes. Prolonged starvation results in decreased levels of *mIndy*, likely due to glucagon’s short half-life. In the midguts of wild-type flies, CR leads to a 50% reduction in *Indy* mRNA compared to its levels on a standard diet (Rogers and Rogina 2014). Furthermore, fly midguts of control flies have increased *Indy* mRNA during aging on a standard diet, when aged on a high-calorie diet or under conditions that mimic aging. It is highly likely that increased *Indy* expression in fly midguts during aging is due to the increased energy needs caused by increased intestinal stem cell (ISC) proliferation. This is supported by the finding that reduced *Indy* activity in heterozygous *Indy* ^*206*^ */+* and *Indy* ^*YC0030*^ */+* flies is associated with reduced ISC proliferation and preservation of ISC function (discussed below) (Rogers and Rogina 2014). In contrast, a high-calorie diet increases *Indy* mRNA expression levels leading to unhealthy conditions. Perhaps not surprising, a direct link was found between increased hepatic *mIndy* levels and human non-alcoholic fatty liver disease (NAFLD) (von Loeffelholz et al., 2017). NAFLD is associated with development of insulin resistance, type 2 diabetes (T2D), and liver cirrhosis (Postic and Girard 2008). *mIndy* expression in the liver was coupled to body mass index (BMI) and to liver fat: reduced *mIndy* levels are associated with low amounts of liver fat in lean subjects, while increased *mIndy* levels were found in subjects with higher BMI and high liver fat. A similar increase in hepatic *mIndy* levels was observed in non-human primates fed a high-fat diet for 2 years. Whole genome transcription analysis of liver samples revealed that NAFLD is associated with increased expression levels of genes involved in lipid metabolism and immunological processes. Furthermore, increased interleukin-6 (IL-6) levels activate transcription of *mIndy* via the transcription factor Stat3 and its binding to two STAT-binding sites in the *Indy* promoter region. Activation of the IL-6-Stat3 pathway increases *mIndy* expression, resulting in increased citrate uptake and increased hepatic lipogenesis *in vivo*. The key experiments that showed a direct link between IL-6 and increased *mIndy* levels were performed in *mINDY* ^*−/−*^ mice, in which stimulatory effects of IL-6 on *mIndy* levels, citrate uptake, and FA synthesis were missing.

Activation of the aryl hydrocarbon receptor (AhR) leads to fatty liver diseases in animal models and in humans. AhR induces *mIndy* expression in rat hepatocytes suggesting that induction of *mIndy* leads to increased levels of cytoplasmic citrate and fat synthesis (Neuschäfer-Rube et al., 2015). A potential AhR binding site was identified in the *mIndy* promoter region, which when eliminated, prevents activation of *mIndy* expression *via* AhR. Similarly, *mIndy* is a transcriptional target for the pregnane X receptor via two enhancer modules upstream of the *mIndy* (*mSLC13A5*) gene transcription start site in human primary liver cells (Li et al., 2015). Activation of the pregnane X receptor by the drug rifampicin activates *mIndy* transcription and lipid accumulation in human hepatocytes (Li et al., 2015).

### *Indy*, Intestinal Stem Cells and Hepatocellular Carcinoma

Intestinal stem cells (ISC) are vital for replacement of damaged cells and maintenance of gut integrity and function. ISC division rate adapts to intestinal growth requirements, damage, stress, as well as nutrients in *Drosophila* and in mammals. While ISCs are essential for replacing damaged cells during aging, continuous exposure to toxins and ROS results in uncontrolled ISC division rate, generating large numbers of ISCs and enteroblasts (EB). This surpasses the differentiation potential of EB and leads to the accumulation of polyploid aggregates that cannot replace damaged cells (Choi et al., 2008; Hochmuth et al., 2011), compromising intestinal integrity. CR maintains ISC homeostasis in older flies and *Indy* reduction is similar to CR in its effects on ISC cells. In flies, *Indy* is strongly expressed on the basolateral membrane of midgut cells. *Indy* reduction (*Indy* ^*206*^ */+* and *Indy* ^*YC0030*^ */+* flies) increases the levels of the transcription factor *dPGC-1/spargel,* resulting in increased mitochondrial biogenesis (Rogers and Rogina 2012; Rogers and Rogina 2014; Rogers and Rogina 2015). Furthermore, these mitochondria produce less ROS, and have increased mitochondrial membrane potential during aging, indicating preserved function (Rogers and Rogina 2014; Rogers and Rogina 2015). INDY reduction also leads to increased levels of antioxidant genes. These physiological changes delay age-associated increases in the ISC proliferation rate, leading to preserved ISC homeostasis (Rogers and Rogina 2014; Rogers and Rogina 2015; Kannan and Rogina 2021). Altogether, these data indicate that *Indy*/INDY expression levels are key for cell proliferation. A similar negative effect upon cell proliferation is seen in human hepatocarcinoma cells when *Indy* gene activity is inhibited, most likely by reducing energy required for cancer cell proliferation (Li et al., 2017; Peters 2017). The authors showed that when mINDY expression was silenced in human hepatocellular carcinoma HepG2 cells, using mINDY-shRNA, xenographs derived from the HepG2 cells did not produce tumors when the cells were injected into nude mice (Li et al., 2017; Peters 2017). Knockdown of mINDY was linked to decreased intracellular citrate levels, reduced ATP/ADP ratio and reduced ATP citrate lyase expression. Remarkably, mINDY reduction resulted in inhibition of oncogenic target of rapamycin signaling *via* activation of the AMP-activated protein kinase. Similarly, application of a mSLC13A5 inhibitor suppressed lipid synthesis, decreased cell viability, ROS production and induced apoptosis of HepG2 cells (Phokrai et al., 2018). These studies demonstrate potential of use of SLC13A5 inhibitors as a novel anti-cancer therapeutic candidate.

### The Role of INDY in Spermatogenesis

A new role for INDY and extracellular citrate in inter-organ communication has been recently described (Hudry et al., 2019). Hudry et al. (2019) showed that intestinal carbohydrate metabolism in flies is sex-dependent. This sex-dependent effect is mediated by the close proximity between the testes and the adjacent intestinal section R4. The testes release the cytokine Upd1, which induces JAK/STAT signaling in enterocytes of the R4 region of the midgut, in turn modulating the expression of genes involved in the handling and metabolism of various sugars, as well as modulating cytosolic citrate production. INDY transports citrate out of the R4 gut cells, allowing citrate uptake in the testes and sperm maturation (Hudry et al., 2019). Genetic studies confirmed that INDY is required for the citrate transport needed for sperm maturation, illustrated by a decrease in spermatocyte numbers in the testes of *Indy* knockout flies (Hudry et al., 2019). It has been suggested that citrate can affect sperm maturation by increasing TCA cycle activity, by acting as a substrate for the fatty acid synthesis (necessary for sperm elongation), and perhaps by affecting histone acetylation via its conversion to acetyl-CoA (Hudry et al., 2019). It would be of interest to examine the role of mINDY in testes of mice, rats and humans, though mINDY is expressed at low levels in mammalian testis (Inoue, et al., 2002a; von Loeffelholz et al., 2017). In contrast, *Indy* ^*206*^ */+* and *Indy* ^*302*^ */+* female flies have increased fecundity compared to controls suggesting sex-specific differences with *Indy* reduction in flies (Rogina et al., 2000; Marden et al., 2003; Kannan and Rogina, 2021). Similar increases in female fecundity were observed in natural populations of flies containing the *Hoppel* transposable element in the *Indy* region (Zhu et al., 2014).

### mINDY and Heart Disease

A recent study in *mINDY* ^*−/−*^ (*mIndy-KO*) mice described a novel role for mINDY in regulating blood pressure via its effects on the sympathoadrenal axis (Birkenfeld et al., 2011; Willmes, et al., 2021). Arterial blood pressure and heart rate were lower in *mINDY* ^*−/−*^ mice, and this effect was mediated by a reduction in catecholamine biosynthesis. *In vivo* findings on the effects of *mINDY* reduction on inhibition of catecholamine biosynthesis were confirmed using mouse pheochromocytoma cells (MPCs), which are derived from the chromaffin cells of the adrenal medulla. Addition of citrate increased catecholamine biosynthesis, while treatment with the mINDY competitive inhibitor PF-0676128 reduced catecholamine biosynthesis, confirming the role of mINDY-mediated transport in regulating catecholamine levels. CR reduces blood pressure by a similar mechanism (Gay et al., 2016). The ability of mINDY to regulate blood pressure and heart rate widens its utility as a target for future therapies.

### Neurological Effects of *mIndy* Reduction in the Mouse

A recent study showed that systemic *mIndy* deletion in *mIndy-KO* mice increased motor coordination and improved social and recognition memory performance (Fan et al., 2021). These mice were obtained from the European Conditional Mouse Mutagenesis Program, and carry a *LacZ* reporter cassette between exons 3 and 4 of the *mIndy* gene and several FRT and loxP sites allowing tissue-specific *mIndy* deletion, which are different from the *mINDY* ^*−/−*^ (*mIndy-KO*) described in Birkenfeld et al. (Birkenfeld et al., 2011; Fan et al., 2021). Identical effects were observed in wild-type mice subjected to CR. There were no additional effects when *mIndy-KO* mice were kept on a CR diet, suggesting a shared mechanism for CR and *mIndy-KO* effects on memory. A similar but lower effect on memory was observed in mice with a nervous system-specific deletion of *mIndy.* The beneficial effect of *Indy* reduction on memory was associated with significantly increased neurogenesis in the hippocampal dentate gyrus, which was previously observed in CR mice. These effects were not found in control mice with liver-specific *mIndy* reduction, illustrating that *mIndy* deletion in the nervous system was required for increased neurogenesis. *mINDY* deletion in the murine nervous system was not associated with epilepsy, and as noted above, these mice had improved memory (Fan et al., 2021). This is in contrast to detrimental phenotypes associated with loss-of-function *mIndy* mutations in humans (discussed more below), which could suggest that INDY transporter characteristics in mice and humans are different. More work will be needed to understand these differences. The original study on *mINDY* ^*−/−*^ mice did not report any epileptic episodes or any behavioral defects (Birkenfeld, et al., 2011). A recent neurological investigation using the same *mINDY* ^*−/−*^ mouse strain showed that the absence of *mIndy* affects citrate levels in cerebrospinal fluid and, in a fraction of the mice, neuronal network excitability in the hippocampus (Birkenfeld, et al., 2011; Henke, et al., 2020). Behavioral tests, video-EEG monitoring, and electrophysiologic studies revealed that a fraction of *mINDY* ^*−/−*^ mice have an increased propensity for epileptic seizures and proepileptic neuronal excitability. Additional proteomic and metabolomic analysis of murine brain and cerebrospinal fluid point to changes in citrate levels as the most likely source of the observed changes (Henke, et al., 2020).

### Neurological Effects of *mIndy* (*mSLC13A5*) Mutations in Humans

Mutations in the coding region of human *mIndy* (*mSLC13A5*), which would result in a loss of function, lead to early infantile epileptic encephalopathy (EIEE) (Thevenon et al., 2014; Hardies et al., 2015; Klotz et al., 2016; Matricardi et al., 2020; Brown et al., 2021). This is a rare autosomal recessive disease that manifests as seizures in children within 24 h of birth, as well as limited speech and motor skills, and developmental delays. Most patients have tooth defects caused by lack of enamel. In addition, patients experience mild non-neurological effects such as gastrointestinal, cardiovascular and respiratory complaints (Brown et al., 2021). Early growth is within the normal range but a few adolescent patients experience slower growth (Brown et al., 2021). Analysis of plasma, cerebrospinal fluid, and urine from EIEE patients revealed elevated levels of plasma citrate and other TCA cycle intermediates (Bainbridge et al., 2017). About one hundred individuals have been diagnosed with EIEE to date, which are caused by about 41 different mutations in the *mIndy* (*mSLC13A5*) gene (Jaramillo-Martinez et al., 2021; Kopel et al., 2021). Mutations have been classified into Class I through III based on the likely impact a mutation might have on protein transport function, protein expression, protein trafficking, and protein stability (Jaramillo-Martinez et al., 2021). Predictions are based on the proposed protein structure of human mINDY. Class I includes missense mutations making the transporter nonfunctional. Class II missense mutations cause protein folding issues leading to defects in membrane trafficking. Class III mutations include those that interfere with protein synthesis, such as introduction of stop codons or nonsense mutations (Duan et al., 2021). Intriguingly, while the original *mINDY* ^*−/−*^ mice show no obvious neuronal dysfunction, video-EEG monitoring and electrophysiologic studies show that a fraction of *mINDY* ^*−/−*^ mice have an increased predisposition for epileptic seizures (Birkenfeld et al., 2011). Furthermore, *mIndy-KO* mice have increased memory suggesting that different *mIndy-KO* models may influence the levels of *mIndy* and the physiological consequences differently (Fan et al., 2021).

Kohlschütter−Tönz syndrome (KTS) is another disease associated with *mIndy* (*mSLC13A5*) mutations and the phenotypes are similar to EIEE (Schossig et al., 2017). The mechanism underlying this severe disorder is unknown, but studies of *mIndy* expression in the brain may provide some insights.

The *mIndy* gene encodes the only known neuronal plasma membrane Na-dependent citrate transporter. RNA sequencing (RNA-seq) data confirm mSLC13A5 expression in the cerebellum, cerebral cortex, hippocampus, and olfactory bulb (Inoue, et al., 2002a; Pajor, et al., 2001). There are several theories as to the causes of epileptic encephalopathy. Cytoplasmic citrate is used for lipid, cholesterol, glucose, and glutamate synthesis, and malate derived from citrate is used for energy production in mitochondria. Some have suggested that mitochondrial dysfunction could lead to a decrease in ATP production in neurons resulting in seizures (Zsurka and Kunz 2015; Bhutia et al., 2017; Kopel et al., 2021). Citrate is also the precursor for several neurotransmitters such as acetylcholine, GABA, and glutamate. Citrate is converted to acetyl CoA, which is then converted into fatty acids and cholesterol that form the myelin sheath in neurons. Zinc is chelated by citrate, which is a known modulator of glutamatergic NMDA receptors. Therefore, some speculate that directly or indirectly, these biological functions of citrate might be affected by mutations in the mINDY transporter function leading to epileptic seizures. While most autosomal recessive mutations in *mIndy* should lead to loss of INDY function, some mutations in the *mIndy* coding region could potentially confer gain-of function phenotypes instead.

### *mIndy-KO* (*mSLC13A5 KO*) Mice and Bone Mineralization

A 2017 study using *mIndy* (*mSLC13A5* ^*−/−*^) deficient C57BL/6 mice demonstrated abnormal tooth enamel formation, bone mineralization, and bone formation at 13 weeks of age (Irizarry et al., 2017). These *mSLC13A5* ^*−/−*^ mice have not been used in other studies. These abnormalities were characterized by discoloration of incisors (which were also easily broken), tooth and mandibular abscesses, and a 14% decrease in bone mineral density of the mid femur, compared with heterozygous and wild-type controls. *mSLC13A5* ^*−/−*^ mice described here had reduced overall body size and decreased body weight compared to controls, which is identical to findings observed in two other *mSLC13A5* ^*−/−*^ *KO* mice models, described above. These mice had no signs of behavioral abnormalities, seizures, or tremors (Irizarry et al., 2017). Abnormalities in tooth and enamel structure were still present at 32 weeks of age, demonstrating the critical role of the citrate transporter in dental development. However, bone density and formation were normal by 32 weeks, suggesting that the citrate transporter may not be as crucial once a mature skeleton has developed. EIEE patients that have biallelic loss of function in *mIndy* exhibit amelogenesis imperfecta, a group of rare inherited disorders associated with abnormal enamel formation (Schossig et al., 2017). Another link between *mIndy* and abnormal bone mineralization was observed in a mouse model of osteogenesis imperfecta (OI): OI mice have 2.5-fold increased levels of *mIndy*, suggesting increased citrate levels cause abnormal mineralization in OI mice bones (Moffatt et al., 2021).

### Small Molecule Inhibitors of INDY (SLC13A5)

**FIGURE 2:**
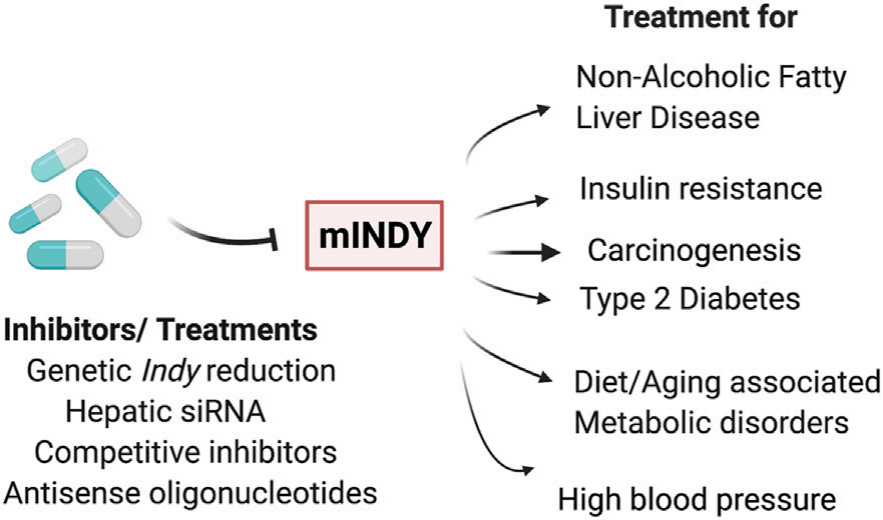
INDY as a therapeutic target: Genetic or pharmacological inhibition of INDY levels/activity has potential to be used for treatment of non-alcoholic fatty liver disease, insulin resistance, carcinogenesis, type 2 diabetes, diet- or aging-induced metabolic disorders, and high blood pressure, as well in carcinogenesis.

Considering the important role of INDY (SLC13A5) in metabolism, INDY has become a particularly attractive target for the treatment of conditions such as obesity, diabetes, and cardiovascular diseases, (Birkenfeld et al., 2011; Rogina 2017; Willmes et al., 2018). A variety of small molecules that inhibit mINDY expression have been described (Huard et al., 2015; Huard et al., 2016; Pajor et al., 2016). For example, PF2 (also known as PF-06649298), a selective Na^+^/citrate transporter (NaCT) inhibitor, has shown high affinity and specificity for mINDY. The interactions of PF2 with mINDY were determined using cryo-EM (Sauer et al., 2021). The protein structure of the NaCT-PF2 complex was identical to the mINDY-citrate complex, confirming the previous notion that PF2 is a competitive inhibitor, which like citrate requires Na^+^ for binding. The PF2 interaction with the scaffold domain blocks the sliding movement of the transport domain required for the transporter to return to the outward facing conformation. In addition, PF2 stops the release of Na^+^ ions leading to inhibition of mINDY (Colas et al., 2015). Weekly injection of liver-specific small interfering RNA (siRNA) against mINDY prevented diet-induced NAFLD and improved hepatic insulin sensitivity in adult C57BL/6J mice fed a Western (high-fat) diet (Brachs et al., 2016). Similarly, second generation antisense oligonucleotides that were targeted to hepatic *mIndy* prevented diet-induced hepatic insulin resistance and hepatic steatosis in rats (Pesta et al., 2015). These rats had similar weight but had reduced fasting plasma insulin levels and, reduced plasma and hepatic triglycerides. Higuchi et al. performed a functional analysis on BI01383298, an irreversible and non-competitive high-affinity inhibitor of human INDY (SLC13A5) (Figure 2) (Higuchi et al., 2020). Promising results were obtained using mINDY as a target in treatment for hepatocellular carcinoma (Li et al., 2017; Peters 2017; Phokrai et al., 2018). HepG2 cells (human hepatocyte carcinoma cells with high proliferation rates) treated with BI01383298 had decreased cell proliferation, consistent with the effects of mINDY inhibition on cell proliferation discussed earlier (Higuchi et al., 2020).

## Concluding Thoughts

**FIGURE 3:**
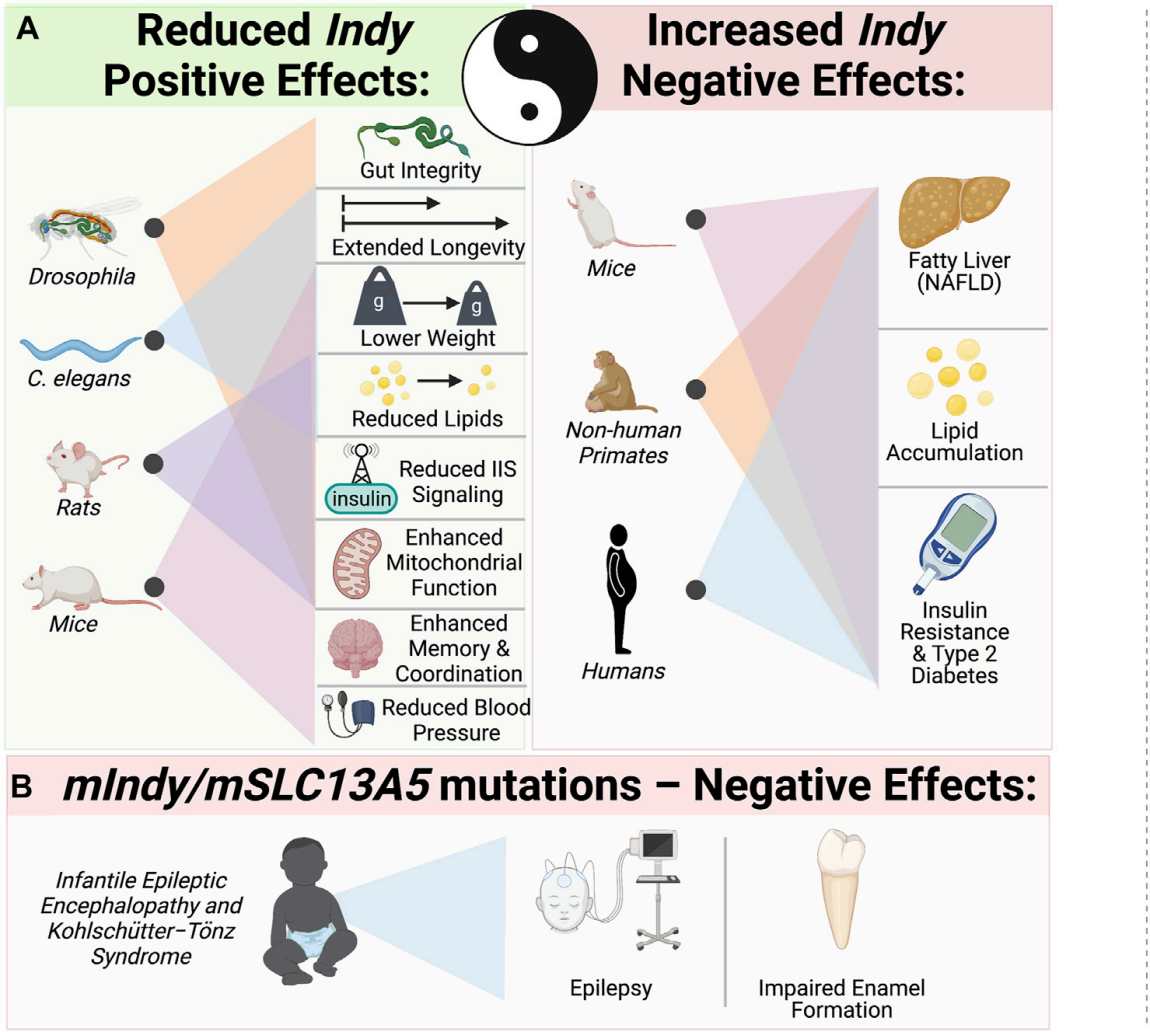
Yin–Yang: The role of INDY in metabolism, health, and longevity. **(A)** Yin - Reduced *Indy* in flies and its homologues in worms extends longevity, lowers weight and reduces lipids. Reduced *Indy* results in reduced IIS, increases mitochondrial biogenesis in flies, mice and rats. *mINDY* ^*−/−*^ have reduced blood pressure and increased memory coordination. Yang–increased *Indy* levels are link to non-alcoholic fatty liver disease (NAFLD), lipid accumulation and insulin resistance in mice, no-human primates on high fat diet and obese insulin resistant humans. **(B)** Mutations in *mIndy (mSLC13A5)* cause autosomal recessive infantile epileptic encephalopathy and Kohlschütter−Tönz syndrome associated with epilepsy, impaired enamel formation and developmental delays.

INDY is a plasma membrane citrate transporter predominantly expressed in metabolically active tissues. Reduction of wild type *Drosophila Indy* and worm homologues extend longevity and have effects on metabolism similar to CR. *mINDY* ^*−/−*^ mice also have metabolic effects that mimics CR. Our group and others have characterized INDY as a physiological regulator, whose expression adapts to the nutrient requirements of cells. INDY’s metabolic effects are a result of its regulation of cytoplasmic citrate, thereby modulating fatty acid synthesis, and glucose metabolism, and energy production in mitochondria. Reduced INDY expression decreases the weight of flies, worms, and mice, and prevents many of the adverse effects of high-fat diets and age on metabolism (Figure 3). Yin and Yang of INDY effects: While low *Indy* levels are associated with increases in overall organismal health, two recent reports link increased *mIndy* levels with NAFLD in an experimental mouse model of NAFLD and human patients with NAFLD (Brachs et al., 2016; von Loeffelholz et al., 2017). The fact that changes in expression of a single gene have strong downstream effects leading to changes in liver metabolism, insulin sensitivity, and development of NAFLD and T2D, opens up the possibility for pharmacological manipulation. Therapeutic potential of competitive stereo-specific inhibitors of the mINDY transporting activity is promising. Temporal administration of these inhibitors to mice reduced lipid levels, increased insulin sensitivity, and protected them from high-fat-diet induced fatty liver disease (Huard, et al., 2015). This underscores the need for further studies on the molecular mechanisms underlying *Indy’s* role in health, disease, and longevity. Of particular interest has been the link between loss-of function mutations in *mIndy* (*mSLC13A5*) and autosomal recessive disorders in children. Taken together, further studies of the *Indy* gene could provide essential insights for effective interventions to promote a healthier and longer life.

## References

[B1] Bainbridge M. N. Cooney E. Miller M. Kennedy A. D. Wulff J. E. Donti T. (2017). Analyses of SLC13A5 -Epilepsy Patients Reveal Perturbations of TCA Cycle. Mol. Genet. Metab. 121, 314–319. 10.1016/j.ymgme.2017.06.009 28673551 PMC7539367

[B2] Bhutia Y. D. Kopel J. J. Lawrence J. J. Neugebauer V. Ganapathy V. (2017). Plasma Membrane Na⁺-Coupled Citrate Transporter (SLC13A5) and Neonatal Epileptic Encephalopathy. Molecules 22, E378. 10.3390/molecules22030378 28264506 PMC6155422

[B3] Birkenfeld A. L. Lee H.-Y. Guebre-Egziabher F. Alves T. C. Jurczak M. J. Jornayvaz F. R. (2011). Deletion of the Mammalian INDY Homolog Mimics Aspects of Dietary Restriction and Protects against Adiposity and Insulin Resistance in Mice. Cel. Metab. 14, 184–195. 10.1016/j.cmet.2011.06.009 PMC3163140 21803289

[B4] Brachs S. Winkel A. F. Tang H. Birkenfeld A. L. Brunner B. Jahn-Hofmann K. (2016). Inhibition of Citrate Cotransporter Slc13a5/MINDY by RNAi Improves Hepatic Insulin Sensitivity and Prevents Diet-Induced Non-alcoholic Fatty Liver Disease in Mice. Mol. Metab. 5, 1072–1082. 10.1016/j.molmet.2016.08.004 27818933 PMC5081411

[B5] Bross T. G. Rogina B. Helfand S. L. (2005). Behavioral, Physical, and Demographic Changes in Drosophila Populations through Dietary Restriction. Aging Cel. 4, 309–317. 10.1111/j.1474-9726.2005.00181.x 16300483

[B6] Brown T. L. Nye K. L. Porter B. E. (2021). Growth and Overall Health of Patients with SLC13A5 Citrate Transporter Disorder. Metabolites 11, 746. 10.3390/metabo11110746 34822404 PMC8625967

[B7] Choi N.-H. Kim J.-G. Yang D.-J. Kim Y.-S. Yoo M.-A. (2008). Age-Related Changes in Drosophila Midgut Are Associated with PVF2, a PDGF/VEGF-like Growth Factor. Aging Cell 7, 318–334. 10.1111/j.1474-9726.2008.00380.x 18284659 PMC2408640

[B8] Colas C. Pajor A. M. Schlessinger A. (2015). Structure-Based Identification of Inhibitors for the SLC13 Family of Na+/Dicarboxylate Cotransporters. Biochemistry 54, 4900–4908. 10.1021/acs.biochem.5b00388 26176240 PMC4781755

[B9] Duan R. Saadi N. W. Grochowski C. M. Bhadila G. Faridoun A. Mitani T. (2021). A Novel Homozygous SLC13A5 Whole‐gene Deletion Generated by Alu/Alu ‐mediated Rearrangement in an Iraqi Family with Epileptic Encephalopathy. Am. J. Med. Genet. 185, 1972–1980. 10.1002/ajmg.a.62192 33797191 PMC8445493

[B10] Fan S.-Z. Sung C.-W. Tsai Y.-H. Yeh S.-R. Lin W.-S. Wang P.-Y. (2021). Nervous System Deletion of Mammalian INDY in Mice Mimics Dietary Restriction-Induced Memory Enhancement. J. Gerontol. A, Biol. Sci. Med. Sci. 76, 50–56. 10.1093/gerona/glaa203 32808644

[B11] Fei Y. J. Liu J. C. Inoue K. Zhuang L. Miyake K. Miyauchi S. (2004). Relevance of NAC-2, an Na+-Coupled Citrate Transporter, to Life Span, Body Size and Fat Content in Caenorhabditis Elegans. Biochem. J. 379 (Pt 1), 191–198. 10.1042/BJ20031807 14678010 PMC1224044

[B12] Ferramosca A. Zara V. (2014). Dietary Fat and Hepatic Lipogenesis: Mitochondrial Citrate Carrier as a Sensor of Metabolic Changes1. Adv. Nutr. 5, 217–225. 10.3945/an.11300476210.3945/an.113.004762 24829468 PMC4013174

[B13] Frankel S. Rogina B. (2012). Indy Mutants: Live Long and Prosper. Front. Gene. 3, 13. 10.3389/fgene.2012.00013 PMC3281209 22363340

[B14] Frankel S. Rogina B. (2021). Evolution, Chance, and Aging. Front. Genet. 12, 733184. 10.3389/fgene.2021.733184 34567083 PMC8458753

[B15] Gay H. C. Rao S. G. Vaccarino V. Ali M. K. (2016). Effects of Different Dietary Interventions on Blood Pressure. Hypertension 67, 733–739. 10.1161/hypertensionaha.115.06853 26902492

[B16] Hardies K. de Kovel C. G. F. Weckhuysen S. Asselbergh B. Geuens T. Deconinck T. (2015). Recessive Mutations inSLC13A5result in a Loss of Citrate Transport and Cause Neonatal Epilepsy, Developmental Delay and Teeth Hypoplasia. Brain 138, 3238–3250. 10.1093/brain/awv263 26384929

[B17] Helfand S. L. Rogina B. (2003a). Molecular Genetics of Aging in the Fly: Is This the End of the Beginning? Bioessays 25, 134–141. 10.1002/bies.10225 12539239

[B18] Helfand S. L. Rogina B. (2003b). Genetics of Aging in the Fruit Fly, *Drosophila melanogaster* . Annu. Rev. Genet. 37, 329–348. 10.1146/annurev.genet.37.040103.095211 14616064

[B19] Henke C. Töllner K. van Dijk R. M. Miljanovic N. Cordes T. Twele F. (2020). Disruption of the Sodium-dependent Citrate Transporter SLC13A5 in Mice Causes Alterations in Brain Citrate Levels and Neuronal Network Excitability in the hippocampus. Neurobiol. Dis. 143, 105018. 10.1016/j.nbd.2020.105018 32682952

[B20] Higuchi K. Kopel J. J. Sivaprakasam S. Jaramillo-Martinez V. Sutton R. B. Urbatsch I. L. (2020). Functional Analysis of a Species-specific Inhibitor Selective for Human Na+-Coupled Citrate Transporter (NaCT/SLC13A5/MINDY). Biochem. J. 477, 4149–4165. 10.1042/bcj20200592 33079129 PMC7657661

[B21] Hochmuth C. E. Biteau B. Bohmann D. Jasper H. (2011). Redox Regulation by Keap1 and Nrf2 Controls Intestinal Stem Cell Proliferation in Drosophila. Cel. Stem Cel. 8, 188–199. 10.1016/j.stem.2010.12.006 PMC3035938 21295275

[B22] Huard K. Brown J. Jones J. C. Cabral S. Futatsugi K. Gorgoglione M. (2015). Discovery and Characterization of Novel Inhibitors of the Sodium-Coupled Citrate Transporter (NaCT or SLC13A5). Sci. Rep. 5, 17391. 10.1038/srep17391 26620127 PMC4664966

[B23] Huard K. Gosset J. R. Montgomery J. I. Gilbert A. Hayward M. M. Magee T. V. (2016). Optimization of a Dicarboxylic Series for *In Vivo* Inhibition of Citrate Transport by the Solute Carrier 13 (SLC13) Family. J. Med. Chem. 59, 1165–1175. 10.1021/acs.jmedchem.5b01752 26734723

[B24] Hudry B. de Goeij E. Mineo A. Gaspar P. Hadjieconomou D. Studd C. (2019). Sex Differences in Intestinal Carbohydrate Metabolism Promote Food Intake and Sperm Maturation. Cell 178, 901–918. 10.1016/j.cell.2019.07.029 31398343 PMC6700282

[B25] Inoue K. Fei Y. J. Huang W. Zhuang L. Chen Z. Ganapathy V. (2002a). Functional Identity of Drosophila Melanogaster Indy as a Cation-independent, Electroneutral Transporter for Tricarboxylic Acid-Cycle Intermediates. Biochem. J. 367 (Pt 2), 313–319. 10.1042/BJ20021132 12186628 PMC1222911

[B26] Inoue K. Zhuang L. Ganapathy V. (2002b). Human Na+-Coupled Citrate Transporter: Primary Structure, Genomic Organization, and Transport Function. Biochem. Biophys. Res. Commun. 299, 465–471. 10.1016/s0006-291x(02)02669-4 12445824

[B27] Inoue K. Fei Y. J. Zhuang L. Gopal E. Miyauchi S. Ganapathy V. (2004). Functional Features and Genomic Organization of Mouse NaCT, a Sodium-Coupled Transporter for Tricarboxylic Acid Cycle Intermediates. Biochem. J. 378 (Pt 3), 949–957. 10.1042/BJ20031261 14656221 PMC1224018

[B28] Irizarry A. R. Yan G. Zeng Q. Lucchesi J. Hamang M. J. Ma Y. L. (2017). Defective Enamel and Bone Development in Sodium-Dependent Citrate Transporter (NaCT) Slc13a5 Deficient Mice. PLoS One 12, e0175465. 10.1371/journal.pone.0175465 28406943 PMC5391028

[B29] Jaramillo-Martinez V. Ganapathy V. Urbatsch I. L. (2021). A home Run for Human NaCT/SLC13A5/INDY: Cryo-EM Structure and Homology Model to Predict Transport Mechanisms, Inhibitor Interactions and Mutational Defects. Bioch. J. 478, 2051–205757. 10.1042/bcj20210211 PMC8203205 34101804

[B30] Kannan K. Rogina B. (2021). The Role of Citrate Transporter INDY in Metabolism and Stem Cell Homeostasis. Metabolites 11, 705. 10.3390/metabo11100705 34677421 PMC8540898

[B31] Klotz J. Porter B. E. Colas C. Schlessinger A. Pajor A. M. (2016). Mutations in the Na(+)/Citrate Cotransporter NaCT (SLC13A5) in Pediatric Patients with Epilepsy and Developmental Delay. Mol. Med. 22, 310–321. 10.2119/molmed.2016.00077 PMC5023510 27261973

[B32] Knauf F. Rogina B. Jiang Z. Aronson P. S. Helfand S. L. (2002). Functional Characterization and Immunolocalization of the Transporter Encoded by the Life-Extending Gene Indy. Proc. Natl. Acad. Sci. 99, 14315–14319. 10.1073/pnas.222531899 12391301 PMC137881

[B33] Knauf F. Mohebbi N. Teichert C. Herold D. Rogina B. Helfand S. (2006). The Life-Extending Gene Indy Encodes an Exchanger for Krebs-Cycle Intermediates. Biochem. J. 397, 25–29. 10.1042/bj20060409 16608441 PMC1479758

[B34] Kopel J. J. Bhutia Y. D. Sivaprakasam S. Ganapathy V. (2021). Consequences of NaCT/SLC13A5/mINDY Deficiency: Good versus Evil, Separated Only by the Blood-Brain Barrier. Biochem. J. 478, 463–486. 10.1042/bcj20200877 33544126 PMC7868109

[B35] Li L. Li H. Garzel B. Yang H. Sueyoshi T. Li Q. (2015). SLC13A5 Is a Novel Transcriptional Target of the Pregnane X Receptor and Sensitizes Drug-Induced Steatosis in Human Liver. Mol. Pharmacol. 87, 674–682. 10.1124/mol.114.097287 25628225 PMC4366797

[B36] Li Z. Li D. Choi E. Y. Lapidus R. Zhang L. Huang S.-M. (2017). Silencing of Solute Carrier Family 13 Member 5 Disrupts Energy Homeostasis and Inhibits Proliferation of Human Hepatocarcinoma Cells. J. Biol. Chem. 292, 13890–13901. 10.1074/jbc.m117.783860 28655760 PMC5566540

[B37] Luckinbill L. S. Clare M. J. (1985). Selection for Life Span in Drosophila Melanogaster. Heredity 55, 9–18. 10.1038/hdy.1985.66 3930429

[B38] Mancusso R. Gregorio G. G. Liu Q. Wang D.-N. (2012). Structure and Mechanism of a Bacterial Sodium-Dependent Dicarboxylate Transporter. Nature 491, 622–626. 10.1038/nature11542 23086149 PMC3617922

[B39] Marden J. H. Rogina B. Montooth K. L. Helfand S. L. (2003). Conditional Tradeoffs between Aging and Organismal Performance of Indy Long-Lived Mutant Flies. Proc. Natl. Acad. Sci. 100, 3369–3373. 10.1073/pnas.0634985100 12626742 PMC152299

[B40] Matricardi S. De Liso P. Freri E. Costa P. Castellotti B. Magri S. (2020). Neonatal Developmental and Epileptic Encephalopathy Due to Autosomal Recessive Variants in SLC13A5 Gene. Epilepsia 61, 2474–2485. 10.1111/epi.16699 33063863

[B41] Moffatt P. Boraschi-Diaz I. Marulanda J. Bardai G. Rauch F. (2021). Calvaria Bone Transcriptome in Mouse Models of Osteogenesis Imperfecta. Ijms 22, 5290. 10.3390/ijms22105290 34069814 PMC8157281

[B42] Mulligan C. Fenollar-Ferrer C. Fitzgerald G. A. Vergara-Jaque A. Kaufmann D. Li Y. (2016). The Bacterial Dicarboxylate Transporter VcINDY Uses a Two-Domain Elevator-Type Mechanism. Nat. Struct. Mol. Biol. 23, 256–263. 10.1038/nsmb.3166 26828963 PMC5215794

[B43] Neretti N. Wang P.-Y. Brodsky A. S. Nyguyen H. H. White K. P. Rogina B. (2009). Long-Lived Indy Induces Reduced Mitochondrial Reactive Oxygen Species Production and Oxidative Damage. Proc. Natl. Acad. Sci. 106, 2277–2282. 10.1073/pnas.0812484106 19164521 PMC2629441

[B44] Neuschäfer-Rube F. Lieske S. Kuna M. Henkel J. Perry R. J. Erion D. M. (2014). The Mammalian INDY Homolog Is Induced by CREB in a Rat Model of Type 2 Diabetes. Diabetes 63, 1048–1057. 10.2337/db13-0749 24222346 PMC3968437

[B45] Neuschäfer-Rube F. Schraplau A. Schewe B. Lieske S. Krützfeldt J. M. Ringel S. (2015). Arylhydrocarbon Receptor-Dependent MIndy (Slc13a5) Induction as Possible Contributor to Benzo[a]Pyrene-Induced Lipid Accumulation in Hepatocytes. Toxicology 337, 1–9. 10.1016/j.tox.2015.08.007 26303333

[B46] Pajor A. M. Gangula R. Yao X. (2001). Cloning and Functional Characterization of a High-Affinity Na+/dicarboxylate Cotransporter from Mouse Brain. Am. J. Physiol.-Cell Physiol. 280, C1215–C1223. 10.1152/ajpcell.2001.280.5.c1215 11287335

[B47] Pajor A. M. de Oliveira C. A. Song K. Huard K. Shanmugasundaram V. Erion D. M. (2016). Molecular Basis for Inhibition of the Na+/Citrate Transporter NaCT (SLC13A5) by Dicarboxylate Inhibitors. Mol. Pharmacol. 90, 755–765. 10.1124/mol.116.105049 27683012

[B48] Parashar V. Rogina B. (2009). DSir2 Mediates the Increased Spontaneous Physical Activity in Flies on Calorie Restriction. Aging 1, 529–541. 10.18632/aging.100061 20157536 PMC2806034

[B49] Pesta D. H. Perry R. J. Guebre-Egziabher F. Zhang D. Jurczak M. Fischer-Rosinsky A. (2015). Prevention of Diet-Induced Hepatic Steatosis and Hepatic Insulin Resistance by Second Generation Antisense Oligonucleotides Targeted to the Longevity Gene MIndy (Slc13a5). Aging 7, 1086–1093. 10.18632/aging.100854 26647160 PMC4712334

[B50] Peters J. M. (2017). Flipping a Citrate Switch on Liver Cancer Cells. J. Biol. Chem. 292, 13902–13903. 10.1074/jbc.h117.783860 28821606 PMC5566541

[B51] Phokrai P. Poolsri W. a. Suwankulanan S. Phakdeeto N. Kaewkong W. Pekthong D. (2018). Suppressed De Novo Lipogenesis by Plasma Membrane Citrate Transporter Inhibitor Promotes Apoptosis in HepG2 Cells. FEBS Open Bio. 8, 986–1000. 10.1002/2211-5463.12435 PMC5986055 29928578

[B52] Postic C. Girard J. (2008). The Role of the Lipogenic Pathway in the Development of Hepatic Steatosis. Diabetes Metab. 34 (6 Pt 2), 643–648. 10.1016/S1262-3636(08)74599-3 19195625

[B53] Rogers R. P. Rogina B. (2012). A Gutsy Way to Extend Longevity. Front. Gene. 3, 108. 10.3389/fgene.2012.00108 PMC3374461 22707956

[B54] Rogers R. P. Rogina B. (2014). Increased Mitochondrial Biogenesis Preserves Intestinal Stem Cell Homeostasis and Contributes to Longevity in Indy Mutant Flies. Aging 6, 335–350. 10.18632/aging.100658 24827528 PMC4032799

[B55] Rogers R. P. Rogina B. (2015). The Role of INDY in Metabolism, Health and Longevity. Front. Genet. 6, 204. 10.3389/fgene.2015.00204 26106407 PMC4460575

[B56] Rogina B. Helfand S. L. (2013). Indy Mutations and Drosophila Longevity. Front. Genet. 4, 47. 10.3389/fgene.2013.00047 23580130 PMC3619052

[B57] Rogina B. Benzer S. Helfand S. L. (1997). Drosophila Drop-Dead Mutations Accelerate the Time Course of Age-Related Markers. Proc. Natl. Acad. Sci. 94, 6303–6306. 10.1073/pnas.94.12.640310.1073/pnas.94.12.6303 9177212 PMC21044

[B58] Rogina B. Reenan R. A. Nilsen S. P. Helfand S. L. (2000). Extended Life-Span Conferred by Cotransporter Gene Mutations in Drosophila. Science 290, 2137–2140. 10.1126/science.290.5499.2137 11118146

[B59] Rogina B. (2017). INDY-A New Link to Metabolic Regulation in Animals and Humans. Front. Genet. 8, 66. 10.3389/fgene.2017.00066 28596784 PMC5442177

[B60] Sauer D. B. Song J. Wang B. Hilton J. K. Karpowich N. K. Mindell J. A. (2021). Structure and Inhibition Mechanism of the Human Citrate Transporter NaCT. Nature 591, 157–161. 10.1038/s41586-021-03230-x 33597751 PMC7933130

[B61] Schlessinger A. Sun N. N. Colas C. Pajor A. M. (2014). Determinants of Substrate and Cation Transport in the Human Na+/Dicarboxylate Cotransporter NaDC3. J. Biol. Chem. 289, 16998–17008. 10.1074/jbc.m114.554790 24808185 PMC4059142

[B62] Schossig A. Bloch-Zupan A. Lussi A. Wolf N. I. Raskin S. Cohen M. (2017). SLC13A5is the Second Gene Associated with Kohlschütter-Tönz Syndrome. J. Med. Genet. 54, 54–62. 10.1136/jmedgenet-2016-103988 27600704

[B63] Schwarz F. Karadeniz Z. Fischer-Rosinsky A. Willmes D. M. Spranger J. Birkenfeld A. L. (2015). Knockdown of Indy/CeNac2 Extends Caenorhabditis Elegans Life Span by Inducing AMPK/Aak-2. Aging 7, 553–567. 10.18632/aging.100791 26318988 PMC4586101

[B64] Thevenon J. Milh M. Feillet F. St-Onge J. Duffourd Y. Jugé C. (2014). Mutations in SLC13A5 Cause Autosomal-Recessive Epileptic Encephalopathy with Seizure Onset in the First Days of Life. Am. J. Hum. Genet. 95, 113–120. 10.1016/j.ajhg.2014.06.006 24995870 PMC4085634

[B65] Toivonen J. M. Walker G. A. Martinez-Diaz P. Bjedov I. Driege Y. Jacobs H. T. (2007). No Influence of Indy on Lifespan in Drosophila after Correction for Genetic and Cytoplasmic Background Effects. PLOS Genet. 3, e95. 10.1371/journal.pgen.0030095 17571923 PMC1892600

[B66] Ugur B. Chen K. Bellen H. J. (2016). *Drosophila* Tools and Assays for the Study of Human Diseases. Dis. Model. Mech. 9, 235–244. 10.1242/dmm.023762 26935102 PMC4833332

[B67] von Loeffelholz C. Lieske S. Neuschäfer-Rube F. Willmes D. M. Raschzok N. Sauer I. M. (2017). The Human Longevity Gene Homolog INDY and Interleukin-6 Interact in Hepatic Lipid Metabolism. Hepatology 66, 616–630. 10.1002/hep.29089 28133767 PMC5519435

[B68] Wang P.-Y. Neretti N. Whitaker R. Hosier S. Chang C. Lu D. (2009). Long-Lived Indy and Calorie Restriction Interact to Extend Life Span. Proc. Natl. Acad. Sci. 106, 9262–9267. 10.1073/pnas.0904115106 19470468 PMC2685744

[B69] Willmes D. M. Kurzbach A. Henke C. Schumann T. Zahn G. Heifetz A. (2018). The longevity gene INDY ( I 'm N ot D ead Y et) in metabolic control: Potential as pharmacological target. Pharmacol. Ther. 185, 1–11. 10.1016/j.pharmthera.2017.10.003 28987323

[B70] Willmes D. M. Daniels M. Kurzbach A. Lieske S. Bechmann N. Schumann T. (2021). The Longevity Gene mIndy (I'm Not Dead, yet) Affects Blood Pressure through Sympathoadrenal Mechanisms. JCI Insight 6, 136083. 10.1172/jci.insight.136083 33491666 PMC7934862

[B71] Zhu C.-T. Chang C. Reenan R. A. Helfand S. L. (2014). Indy Gene Variation in Natural Populations Confers Fitness Advantage and Life Span Extension through Transposon Insertion. Aging 6, 58–69. 10.18632/aging.100634 24519859 PMC3927810

[B72] Zsurka G. Kunz W. S. (2015). Mitochondrial Dysfunction and Seizures: The Neuronal Energy Crisis. Lancet Neurol. 14, 956–966. 10.1016/s1474-4422(15)00148-9 26293567

